# The Path of Glory Leads but to the Grave

**Published:** 1902-02

**Authors:** 


### Abstracts and Selections.

### "The Path of Glory Leads but to the Grave."

At a banquet given to the Brazos Valley Medical Association at Calvert, the following toast was given: "The path of glory leads but to the grave." The response was made by Dr. M. K. Lott, of Cameron.

Dr. Lott said:

*"Mr. Toastmaster, Gentlemen of the Brazos Valley Medical Association, Ladies and Gentlemen:*

"'The path of glory leads but to the grave.' In some respects this is a fitting toast at a banquet of doctors. Graveyards and doctors are often associated in the mind—to mention one is to think of the other. There is not a house in the civilized world where doctors are not employed; there is not a house in the world where there is not a vacant chair whose occupant has not trod the path that leads to the grave. Sometimes in my efforts to cure disease I have not been so sure that I had not held the lamp that illuminated for the sick the path that leads to the grave. Sometimes I have heard the suggestion that such was the case. Generally such unkind insinuations from some charitable brother doctor (with accent on charitable), who happened not in the case to have held one of the lamps that lighted the path of glory that led the patient to the grave. It is understood that such insinuations never came from members of the Brazos Valley Medical Association—present company is always excepted.

"I have seen the path of glory end at the grave, for many a noble, big hearted physician was his monument measured by his life, his sacrifices, his charity, his broad humanity, a lamp upon its summit would light a path of glory reaching to the skies, and yet when the golden bowl of life was broken his path of glory ended at the grave.

"But recently it has been ascertained both in England and America that in building monuments for those giants in mind and heart, who have signalized their day and generation, who have trodden paths of glory that lead beyond the grave, though hundreds of names have been suggested as entitled to imperishable glory the names of but few physicians were on the list. Why is this?

"The military heroes weave for themselves in the hearts of a grateful nation wreathes of immortal glory and enshrine their names in imperishable splendor on the tablets of fame. The path for Lee and Jackson did not end at the grave, but it leads on and on in a blaze of glory whose effulgence shall forever dazzle this world.

"The poet and the bard are beyond the tooth of time. Thackeray, Dickens, Darwin, Spencer, have marked for themselves paths of glory that lead beyond the grave and that will keep alive their names forever, long after the life and character of the most distinguished physician has faded away and been forgotten. The reason for this is not hard to find. The average doctor is so ingrossed in the service of the distressed and the diseased, striving to the uttermost of his ability to save life or support it where he cannot save, that he neglects his own path of glory, and at the grave it ends.

" 'The boast of heraldry, the pomp of power,
And all that beauty, all that wealth e'er gave,
Await alike the inevitable hour;
The path of glory leads but to the grave.'

"When Sir John Wolfe, before Quebec, quoted this verse and said he had rather be its author than to be the victor in that battle, he well knew that in the 'Elegy in a Country Church Yard' the immortal Gray had blazed for himself a path of glory. Gray, in the sentence contained in this toast, did not refer to the master spirits in the world's history, in the arts and sciences, in story and song, who have traveled paths of glory that lead beyond the grave.

"Ladies and gentlemen, when the Father of Medicine, four hundred years before the Christian era, announced the great truth of the *vis medicatrix naturaæ,* a doctrine of which the medical world has never since lost sight and penned the immortal oath that bears his name, Hyppocrates trod a path of glory that leads beyond the grave.

"When Galen, in the second century of the Christian era, wrote his history of medicine and placed in that book all that was then known of medicine and surgery, he blazed for himself a path of glory that led beyond the grave.

"And in the sixteenth century of the Christian era, when Ambrose Paré, that great French surgeon, applied for the first time a silken ligature to a bleeding blood vessel and arrested the crimson current as it ebbed life away, he walked a path of glory that has led beyond the grave.

"William Harvey, in the seventeenth century, after years of patient toil and study in the dead house among those whose paths of life had ended there, gathering the rays of light that had illuminated the paths of glory of other physiologists that had ended at the grave, he added to them the incandescent light of his own matchless genius—he laid bare forever the mechanism of the circulation of the blood; he trod a path of glory that has led beyond the grave.

"Sir Edward Jenner for twenty years industriously studied the physical characteristics on the hands of English milkmaids, and announced the doctrine of vaccination that has preserved the beauty and vitality not only of a nation, but of the world; has led to the discoveries of Pasteur, Koch, Loeffler and others, establishing on scientific basis the antitoxin treatment of consumption, diphtheria, scarlatina and other contagious diseases hitherto of the highest mortality, and that annually robbed the homes of the world of thousands of the sweet buds that bloom in the hearts of loving mothers and fond fathers, he grandly strode a path of glory that has led beyond the grave.

"When Ephram McDowell, in the wilds of a Kentucky forest an hundred years ago, dared defy a threatening mob of his patient's friends, opened the hitherto unexplored abdominal cavity and successfully removed therefrom an ovarian tumor and saved the life of his patient that must soon have perished, he gave the world ovariotomy and preserved the lives of the blessed mothers of this land—he mapped out for himself a path of glory that has led beyond the grave.

"But why single a few names, when even in the United States there is a galaxy of great men, whose names resound from Boston to Texas? But I shall mention but two, and they belong to our own State. They were ours.

"When, in 1878, the cry of distress went up from yellow fever-stricken Memphis, Grenada and Holly Springs, that cry was heard in Austin. Dr. T. D. Manning, in the bloom of youth, at the zenith of a lucrative and successful practice—everything to lose, nothing to gain, for he had not had the disease. His life had but kissed the rising sun of a glorious morning, when to him every ripple of the brook was a melody, every note of the bird was a song, every flower that blossemed was a poem of love. He had but to turn a deaf ear to the cry for help from these stricken cities and live on in the enjoyment of the rich gifts his fine professional attainments vouched to him. But his great heart was moved in sympathy by the cry of distress, and he went there to do and to die for suffering humanity. When that young life went out it winged its flight to its God along a path of glory that led beyond the grave.

And his friend, his companion, who went with him on that mission of mercy? To mention his name is to be on sacred ground and in touch with a sentiment that weeps but is not weak; commanding an humble reverence that walks barefoot with uncovered head in its presence.

" 'Green be the turf upon thy grave,
Friend of my early days;
None knew thee but to love thee,
None named thee but to praise.'

The splendid man, the superb knightly soldier, the accomplished physician and sanitarian, the noble, generous friend, the devoted husband and father whom to know was to love—Dr. R. M. Swearingen. When he knew for himself life's fitful fever would soon be o'er, with feeble hand and in pencil he wrote on his will in which he bequeathed the fruits of his industrious life to those whom he loved, 'Without any ill feeling towards any being in the world, without fear, I walk out into the great unknown.' It was a long path of glory that led beyond the grave.

The graves of such as these are kept forever green, watered from the fountains of gratitude and of love.

"'When Spring, with dewy fingers cold,
Returns to deck the hallowed mold,
They there shall dress a sweeter sod
Than Fancy's feet have ever trod.
By forms unseen their knell is rung,
By tongues unheard their dirge is sung;
Then Honor comes, a pilgrim grey,
To deck the turf that tops through clay,
And Freedom shall awhile repair,
To dwell a weeping hermit there.'"

